# Venous thromboembolism and hyperhomocysteinemia as first manifestation of pernicious anemia: a case series

**DOI:** 10.1186/s13256-017-1415-z

**Published:** 2017-09-02

**Authors:** W. Ammouri, Z. Mezalek Tazi, H. Harmouche, M. Maamar, M. Adnaoui

**Affiliations:** Internal Medicine Department, Ibn Sina Hospital, University Mohamed V of Medicine, Rue Lamfadel Cherkaoui, BP 6527 Rabat, Morocco

**Keywords:** Homocysteine, Pernicious anemia, Venous thrombosis, Cobalamin

## Abstract

**Background:**

Hyperhomocysteinemia has been suspected of favoring thrombosis. Several case–control studies and even a meta-analysis have confirmed a link between venous thrombosis and hyperhomocysteinemia. Homocysteine is due to genetic and acquired factors (poor diet in folate and vitamin B12, older age, renal impairment, thyroid diseases, and malignancies) induced by the intake and the concentrations of vitamin B9 or B12 in the majority of cases.

**Cases presentation:**

We report the cases of four Moroccan patients who presented with acute vein thrombosis of different sites: a 34-year-old man, a 60-year-old man, a 58-year-old man, and a 47-year-old woman. All patients had a low level of cobalamin with marked hyperhomocysteinemia with normal serum and red cell folic acid. Venous thrombosis revealed pernicious anemia in all patients. Their low levels of cobalamin, atrophic gastritis, and positive results for gastric parietal cell antibodies confirmed the diagnosis of pernicious anemia. There was no evidence of immobilization, recent surgery, malignancy, antiphospholipid antibody, myeloproliferative disorder, or hormone replacement therapy. No deficiencies in protein C and protein S were detected; they had normal antithrombin III function and factor V Leiden; no prothrombin gene mutations were detected. Treatment included orally administered anticoagulation therapy and cobalamin supplementation. The outcome was favorable in all cases.

**Conclusions:**

These reports demonstrate that pernicious anemia, on its own, can lead to hyperhomocysteinemia that is significant enough to lead to thrombosis. Understanding the molecular pathogenesis of the development of thrombosis in patients with hyperhomocysteinemia related to Biermer disease would help us to identify patients at risk and to treat them accordingly. The literature concerning the relationship between homocysteine and venous thrombosis is briefly reviewed.

## Background

Homocysteine is an amino acid formed from the intracellular demethylation of methionine. Hyperhomocysteinemia is characterized by an elevation of serum homocysteine levels. It is thought to be a modifiable risk factor of myocardial infarction, peripheral arterial thrombosis, as well as deep vein thrombosis and pulmonary embolism [1–3]. Most reports related to arterial disease describe an association with mildly increased homocysteine level. By contrast, there are limited and conflicting publications related to venous system thrombosis associated with homocysteine level [4–8].

Hyperhomocysteinemia may result from genetic defects in the enzymes involved in homocysteine metabolism: cystathionine ß-synthase (CBS), methionine synthase (MS), and N5,N10-methylenetetrahydrofolate reductase (MTHFR) or from deficiencies of enzymes cofactors (vitamin B6, vitamin B12, or cosubstrate vitamin B9) [5].

However, the most common cause of vitamin B12 deficiency with hyperhomocysteinemia is pernicious anemia. Pernicious anemia is usually diagnosed in the presence of megaloblastic anemia, neurologic symptoms, or atrophic gastritis. Thrombotic events have been reported to be a revealing symptom [9–16]. We reported four cases of venous thrombosis revealing pernicious anemia.

## Case presentation

### Case 1

A 34-year-old Moroccan man was admitted to our intensive care unit because of dyspnea. He had been under treatment for psychosis for 3 years. His physical examination was normal. There was no physical sign of thrombophlebitis. Chest radiographs and an electrocardiogram were unremarkable. His hemoglobin was 9 g/dl; his mean corpuscular volume was 120 μm^3^. His prothrombin time, partial thromboplastin time, and fibrinogen level were normal. A spiral computed tomography scan of his chest revealed bilateral pulmonary embolism. There was no clinical or biological evidence of neoplasia, Behçet disease, antiphospholipid syndrome, or systemic lupus. He also had a normal platelet count, normal protein C and protein S levels, and normal antithrombin III function. Genetic testing for factor V Leiden and factor II mutation was negative. His plasma homocysteine level was 50 μmol/l (normal < 16) and cobalamin plasma level was measured at 60 pg/ml (normal > 120). His folate plasma level was normal. Antibodies to intrinsic factor were positive. Bone marrow aspiration with biopsy showed megaloblastosis. An endoscopy revealed atrophic gastritis. Treatment included orally administered anticoagulation therapy and cobalamin supplementation, initially parenteral. After a 1-year follow-up period, he remained free of psychiatric disorders and thrombotic events. His hemoglobin and homocysteine plasma levels were within normal range.

### Case 2

A previously healthy 60-year-old Moroccan man without any medical history presented to our hospital with anemia and a deep venous thrombosis in his right leg. A physical examination showed pallor and swelling of his right leg with signs of phlebitis. Ultrasonography revealed thrombophlebitis in his right ileofemoral and popliteal veins. His hemoglobin level was 9.5 g/dl and his mean corpuscular volume was 111 μm^3^. His plasma homocysteine level was 125 μmol/l (normal < 15) and cobalamin plasma level was 60 pg/ml (normal > 120). His folate plasma level was within the normal range. Bone marrow aspiration with biopsy showed megaloblastosis. Antibodies to intrinsic factor were positive; an endoscopy revealed atrophic gastritis. No other abnormality was found in a more detailed screen for neoplasm or Behçet disease. Antinuclear antibody was negative.

His treatment included intravenously administered anticoagulant therapy with heparin, which was later administered orally. Vitamin B12 was given at high dose intravenously. At day 7, his hemoglobin was 11 g/dl.

After a 6-month follow-up period, under cobalamin and orally administered anticoagulant treatment, his hemoglobin and homocysteine plasma levels were within normal range. He remained free of thrombotic events for 3 years after the follow-up.

### Case 3

**Fig. 1:**
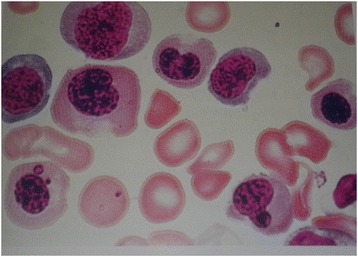
Bone marrow aspiration showed megaloblastosis

A 58-year-old Moroccan man presented to our hospital with anemia and a swelling of his right leg. He had no medical history and a physical examination showed pallor and signs of phlebitis in his right leg. Ultrasonography revealed a thrombophlebitis in his right femoral and popliteal veins. His hemoglobin level was 8.6 g/dl and his mean corpuscular volume was 115 μm^3^. His plasma homocysteine level was 200 μmol/l (normal < 15), his cobalamin plasma level was 60 pg/ml (normal > 120), and his folate plasma level was normal. Bone marrow aspiration with biopsy showed megaloblastosis (Fig. 1). Antibodies to gastric parietal cells were positive, while antibodies to intrinsic factor were normal; an endoscopy revealed fundal atrophic gastritis. No other abnormality was found in a more detailed screen for neoplasm or Behçet disease. Antinuclear antibody tests were negative.

His treatment included intravenously administered anticoagulant therapy with heparin, which was later administered orally. Vitamin B12 was given at a high dose intravenously. At day 15, his hemoglobin was 10 g/dl. After a 6-month follow-up period, under cobalamin and orally administered anticoagulant treatment, his hemoglobin and homocysteine plasma levels were within normal range. He remained free of thrombotic events during 4 years of follow-up.

### Case 4

A 47-year-old Moroccan woman presented to our hospital unit with pain and swelling to her left leg. She had been depressed and had a lability of mood for 1 year and a history of myocardial ischemic stroke 2 years earlier.

A physical examination showed signs of phlebitis in her left leg. Chest radiographs and an electrocardiogram were unremarkable. Her hemoglobin was 12 g/dl; her mean corpuscular volume was 85 μm^3^. Her prothrombin time, partial thromboplastin time, and fibrinogen level were normal.

Ultrasonography revealed thrombophlebitis in her left popliteal vein. There was no clinical or biological evidence of neoplasia, Behçet disease, antiphospholipid syndrome, thrombophilic abnormality, or systemic lupus. Her plasma homocysteine level was 167 μmol/l (normal < 16) and her cobalamin plasma level was measured at 21 pg/ml ( >120). Her folate plasma level was normal. She also had a normal platelet count, normal protein C and protein S levels, and normal antithrombin III function. Genetic testing for factor V Leiden mutation and factor II mutation was negative. Antibodies to intrinsic factor were positive. An endoscopy revealed chronic gastritis and the presence of *Helicobacter pylori*. No other abnormality was found in a more detailed screen for neoplasm or Behçet disease. Treatment included orally administered anticoagulation therapy and cobalamin supplementation, initially parenteral. After a 3-year follow-up period, she remained free of psychiatric disorders and thrombotic events. Her homocysteine plasma level was within normal range.

## Discussion

We reported four cases of pernicious anemia revealed by venous thrombosis secondary to hyperhomocysteinemia. In the three first cases, the diagnostic of pernicious anemia was suspected and then confirmed on the basis of the biological findings: macrocytosis, cobalamin deficiency, bone marrow aspiration with biopsy, gastric atrophy, and intrinsic factor antibodies. However, in case 4, a bone marrow aspiration with biopsy was not done because there was no hematological abnormality. But we had tested for cobalamin deficiency and then for hyperhomocysteinemia because she had psychiatric disorders and a history of ischemic myocardial stroke. All patients presented with severe clinical manifestations at admission. All of our patients had very high levels of plasma homocysteine (50 to 200 μmol/l). The hyperhomocysteinemia secondary to pernicious anemia may explain the thrombosis in our observations. Also, all of our cases had no other risk factors for vascular thrombosis: no clinical or biological evidence of neoplasia, Behçet disease, antiphospholipid syndrome, thrombophilic abnormality, or systemic lupus. After vitamin supplementation, the biological abnormalities disappeared and the follow-up was free of vascular thrombotic events in all patients with a median follow-up of 3 years.

The first case is original in that psychiatric manifestations and pulmonary embolism were the first manifestations of hyperhomocysteinemia-related pernicious anemia. After vitamin B12 supplementation, his clinical and biological abnormalities disappeared.

In case 4, ischemic myocardial stroke and depression were probably the first manifestations of hyperhomocysteinemia related to pernicious anemia. Also, cases 1 and 4 had psychiatric disorders which resolved completely after vitamin supplementation. In the literature, psychiatric manifestations are frequently associated with pernicious anemia including depression, mania, psychosis, and dementia; these manifestations are observed in the absence of other well-recognized neurological and hematological signs (similar to case 4) for months or years [17, 18].

Homocysteine is synthesized by two metabolic pathways: remethylation and transsulfuration. These pathways require vitamin B12 and folate for methionine synthesis and pyridoxal-5 phosphate for cystathionine synthesis [5].

Hyperhomocysteinemia may be related to genetic defects, such as CBS deficiency or thermolabile variant of MTHFR, or to deficiencies of related B group vitamins.

It was thought that the main pathophysiological link among these vitamins and venous thrombosis is the accumulation of homocysteine due to decreased concentrations of these B group vitamins. However, all of these vitamins have a homocysteine-independent role related to the development of venous thrombosis. In addition, hyperhomocysteinemia inhibits the inactivation of factor Va by activated protein C and could increase the effect of factor V Leiden [6, 19].

Severe hyperhomocysteinemia (> 100 μmol/L) is most often caused by CBS deficiency. Mild or moderate hyperhomocysteinemia may result from a relative deficiency of folic acid and vitamin B12 and homozygosity for the common polymorphism 677CT in the *MTHFR* gene [20].

Inherited metabolic abnormality may be suspected when patients present with recurrent episodes of thromboembolism events that occur at an early age or thrombosis at unusual sites.

Cases 2, 3, and 4 presented with severe hyperhomocysteinemia. We suspected a genetic mutation of MTHFR or CBS or other genetic mutations, but these tests were not available in our hospital. However, it can be assumed that our patients did not have these deficits given their clinical presentation and favorable outcome under B12 supplementation alone.

CBS deficiency is characterized by lens dislocation, skeletal abnormalities, neurologic disturbances, and thromboembolism. MTHFR deficiency leads to various neurological symptoms, ranging from developmental delay to encephalopathy, including motor and gait abnormalities, seizures, psychiatric manifestations and, rarely, strokes. The treatment of CBS depends on vitamin B6, whereas MTHFR deficiency can be efficiently treated by vitamin B12, folic acid, and betaine [5, 20].

A study using data from the National Health and Nutrition Examination Survey (NHANES) between 1999 and 2002 found that participants with vitamin B12 deficiency and high serum folate had increased homocysteine levels compared to participants who had the combination of vitamin B12 deficiency and low serum folate, suggesting a role for folate levels in the enzymatic functions of vitamin B12 [20].

In our cases, we speculate that normal folate levels may have contributed to the delay in diagnosing pernicious anemia leading to severe hyperhomocysteinemia and the consequent development of vascular injury and hypercoagulability. However, the absence of family histories for atherothrombotic diseases together with the normalization of their homocysteine levels after parenteral vitamin B12 supplementation strongly suggest that vitamin B12 malabsorption secondary to pernicious anemia is the underlying cause of severe hyperhomocysteinemia.

Vitamin B12, folic acid, and vitamin B6 deficiency are associated with variable elevation of homocysteine levels [5, 21]. It remains unclear whether hyperhomocysteinemia of different causes entails the same risk of thrombosis.

Many hypotheses have been suggested to explain how hyperhomocysteinemia may lead to venous thrombosis. One hypothesis is that homocysteinemia has a toxic effect on the vascular endothelium and on the dotting cascade [1]. Also, homocysteine has several procoagulant properties including the decrease of antithrombin III binding to endothelial heparan sulfate, increase of affinity between lipoprotein(a) and fibrin, induction of tissue factor activity in endothelial cells, and inhibition of inactivation of factor V by activated protein C [22, 23].

Several clinical studies reported more prevalent increase in homocysteinemia in patients with venous thrombosis than in controls [24]. However, the association between hyperhomocysteinemia and venous thrombosis remains controversial. Multivariate analyses of a case–control study showed that low methionine concentration and low methylfolate in red blood cells but not homocysteine were risk factors of venous thrombosis, suggesting that homocysteine is only a marker of vitamin deficiency [21]. Brattström *et al*. [25] found no significant differences in plasma homocysteine concentration between healthy control participants and patients with venous thromboembolism (VTE). Falcon *et al*. [26] reported a high prevalence of hyperhomocysteinemia in patients less than 40 years of age who had VTE. Ducros *et al*. [7] showed that mild or moderate hyperhomocysteinemia does not seem to be a strong determinant in VTE. Also, Ekim *et al*. [6] found a prevalence of hyperhomocysteinemia (15%) in patients with deep venous thrombosis. In this study, there were 26 patients (43.3%) with low folate concentration, of whom five had hyperhomocysteinemia. This study implies that hyperhomocysteinemia and low folate intake may be a risk factor for deep venous thrombosis.

Nevertheless, it is not known whether homocysteine-lowering therapy such as folic acid, vitamin B6, or vitamin B12 supplementation can modify the thrombogenic potential of hyperhomocysteinemia in prevention of recurrent venous thrombosis [27].

Despite the low correlation between hyperhomocysteinemia and cobalamin deficiency, and hyperhomocysteinemia and thrombosis, at least 20 case reports of venous thrombosis in the setting of vitamin B12 deficiency have been published in the literature. The male predominance is remarkable. In many cases hyperhomocysteinemia secondary to pernicious anemia was revealed by a thrombosis. In addition to the case of our first patient, only five cases [10, 13, 14, 28, 29] of pernicious anemia revealed by pulmonary embolism have been published.

Also, other associated hypercoagulation states were reported, comprising anticardiolipin antibodies, factor II mutation, HIV infection with increased fibrinogen, factor VIII mutation, and oral contraception. The mechanism of interaction between homocysteine and other thrombophilic factors is not clear. In all reported cases, homocysteine and cobalamin levels normalized after cobalamin therapy without recurrence of thrombotic events.

However, lowering mildly elevated homocysteine levels in patients with and without vascular disease using vitamin supplementation did not show a reduction in cardiovascular events in several prospective and randomized clinical studies [30–32]. Severe elevation in homocysteine level should be corrected with appropriate vitamin therapy to prevent vascular complications [29].

## Conclusions

These cases demonstrate that vitamin B12 deficiency caused by pernicious anemia may lead to a severely elevated homocysteine level that can be rapidly corrected with vitamin B12 supplements and prevent thrombotic events from recurring. Thus, these conditions should remain in the clinician’s mind, especially when thrombosis occurs along with biological abnormalities such as anemia, megaloblastosis, or hemolysis.

## Abbreviations

CBS: Cystathionine ß-synthase
MS: Methionine synthase
MTHFR: Methylenetetrahydrofolate reductase
NHANES: National Health and Nutrition Examination Survey
VTE: Venous thromboembolism
